# Levator Ani Deficiency and Pelvic Floor Dysfunction 1 Year Postpartum: A Prospective Nested Case–Control Study

**DOI:** 10.1111/1471-0528.18036

**Published:** 2024-12-03

**Authors:** Markus Harry Jansson, Sophia Brismar Wendel, Emilia Rotstein

**Affiliations:** ^1^ Department of Clinical Sciences Danderyd Hospital, Karolinska Institute Stockholm Sweden; ^2^ School of Medical Sciences, Faculty of Health and Medicine Örebro University Örebro Sweden; ^3^ Department of Obstetrics and Gynaecology Danderyd Hospital Stockholm Sweden; ^4^ Department of Clinical Science, Intervention and Technology Karolinska Institute Stockholm Sweden; ^5^ Karolinska Pelvic Floor Centre, Department of Gynaecology and Reproductive Medicine Karolinska University Hospital Stockholm Sweden

**Keywords:** LAD score, levator ani avulsion, levator ani muscle, pelvic floor dysfunction, ultrasound, vaginal birth

## Abstract

**Objective:**

First, to assess whether levator ani deficiency (LAD) is associated with pelvic floor dysfunction 1 year postpartum, including urinary, vaginal and bowel symptoms; and second, to explore at what cut‐off of LAD score such pelvic floor dysfunction arises.

**Design:**

Nested case–control study.

**Setting:**

Örebro University Hospital, Örebro, Sweden.

**Population or Sample:**

Primiparous women 1 year after vaginal birth.

**Methods:**

Three‐dimensional endovaginal ultrasound assessment of the levator ani muscle; LAD score based on this ultrasound, and validated questions about pelvic floor dysfunction. Logistic regression models were used.

**Main Outcome Measures:**

Symptoms of pelvic floor dysfunction associated with LAD.

**Results:**

Altogether 190 women were included, 103 of whom were symptomatic cases and 87 asymptomatic controls. 53% in the case group, and 58% in the control group had a LAD score of 0. A greater LAD score was significantly associated with urinary incontinence (adjusted odds ratio [aOR] 1.11, 95% confidence interval [CI] 1.00–1.22) and vaginal laxity (aOR 1.14, 95% CI 1.03–1.25). The risk of urinary incontinence was increased when the LAD cut‐off score was set between ≥ 1 point and ≥ 4 points. The risk of vaginal laxity was increased when the cut‐off was set between ≥ 8 and ≥ 14 points.

**Conclusions:**

LAD was associated with both urinary incontinence and vaginal laxity. The risk of urinary incontinence increased already with minor LAD and defects of the most medial levator ani muscle portions normally supporting the midurethra may explain this increase.

## 1 Introduction

The levator ani muscle has a complex anatomy tightly linked to its function. Advances in magnetic resonance imaging (MRI) and three‐dimensional (3D) ultrasound during the last two decades have allowed the levator anatomy to be visualised in detail [1, 2]. From the midline and out, the pubococcygeal component has been found to include pubovaginalis, puboperinealis and puboanalis portions supporting the urethra and vagina, perineum and anal canal, respectively [3]. Dorsolateral to the pubococcygeal component, the iliococcygeal component forms a floor supporting the pelvic organs. The puborectal component closes the levator hiatus by pulling the rectum forward [3]. Various degrees of levator injuries occur in 13%–36% of women during vaginal delivery [4].

Three‐dimensional ultrasound has revolutionised pelvic floor imaging by its ready availability, feasibility and cost efficiency compared with MRI [5]. Endovaginal 3D ultrasound (EVUS) assesses the levator components in detail, which is not the case for transperineal 3D ultrasound (TPUS) used in most ultrasound studies on levator injuries [6]. The extent of levator injury visualised by EVUS is presented in terms of ‘levator ani deficiency’, described as a gradient rather than a dichotomous defect or avulsion, as diagnosed by TPUS [6, 7]. The levator ani deficiency score (LAD score) has shown high interrater agreement and moderate ability to predict levator ani muscle strength [8, 9, 10].

When affected women and healthcare providers were asked to identify and prioritise knowledge gaps in the field of maternal birth injuries, they highlighted an increased understanding of symptoms of levator injuries as important in providing better diagnostics [11]. Several studies have demonstrated the association between levator injuries and anatomical pelvic organ prolapse, but the evidence on the relationship between levator injuries and pelvic floor symptoms is controversial [6, 12]. There is some evidence that levator avulsions are associated with vaginal symptoms, including bulging, vaginal laxity and reduced pelvic floor muscle strength, while the association with urinary and faecal incontinence is unclear [13, 14, 15]. However, very few studies have investigated the association between levator ani deficiency assessed by EVUS, and symptoms of pelvic floor dysfunction (PFD) in general [10, 16]. Moreover, the degree of levator ani deficiency at which PFD other than pelvic organ prolapse will start to show has not been evaluated [6]. Therefore, the aim of this study was twofold: first, to test the hypothesis that levator ani deficiency is associated with PFD 1 year postpartum, including urinary, vaginal and bowel symptoms; and second, to explore the levator ani deficiency cut‐off at which such PFD arises.

## 2 Methods

In this nested case–control study, participants were recruited from the Pelvic Floor in Pregnancy and Childbirth (POPRACT) cohort. The POPRACT study was a prospective cohort study conducted in Region Örebro County, Sweden. The methodology of the POPRACT study has been published previously [17, 18, 19, 20]. In summary, nulliparous women were enrolled in early pregnancy during maternity healthcare visits between 1 October 2014 and 1 October 2017. They completed web‐based questionnaires on four occasions during pregnancy and postpartum. The last questionnaire was sent out 1 year postpartum. Questionnaires included items from validated instruments on PFD (Table S1) [21, 22, 23].

Out of the original POPRACT cohort, women who reported a certain degree of bother or frequency of pelvic floor symptoms at 1 year postpartum (potential cases) were invited to a clinical examination of the pelvic floor, which included EVUS. When inviting a potential case, a woman who recently reported no or mild pelvic floor symptoms at 1 year postpartum was invited to an examination (potential control). If a potential control refused, another woman who reported no or mild pelvic floor symptom in the 1‐year questionnaire was repeatedly invited until a control was recruited, the aim being to achieve an equally large control group (thus controls were unmatched). The following pelvic floor symptoms, with the degree of bother or frequency given in parentheses, served as criteria for being invited to the case group: urinary incontinence (moderately, or quite a bit), urinary incontinence during sexual activity (always, often, or sometimes), fear of urinary or stool incontinence restricting sexual activity (always, often, or sometimes), vaginal bulging (sometimes, or often), need for vaginal digitation or splinting to complete bowel evacuation (moderately, or quite a bit), vaginal chafing (often), need to lift the anterior vaginal wall to start or complete voiding (sometimes, or often) or avoidance of sexual intercourse because of vaginal bulging (always, often, or sometimes), ‘sensation of wide vagina’ (any degree of bother or frequency), incontinence to solid and/or liquid stools (any degree of bother), and incontinence to flatus (moderately or quite a bit). The phrase ‘sensation of wide vagina’ was used as a proxy for the symptom of vaginal laxity. Recent joint reports on terminology recommend using this term and we will therefore refer to it hereafter [24, 25]. Women who had delivered by Caesarean section or were pregnant again, as either reported in the 1‐year questionnaire or established at the examination, were excluded. No core outcome set was available for use when designing the study. Projects to develop core outcome sets for childbirth pelvic floor trauma and pelvic floor disorders, respectively, have been presented but have not yet been completed [26, 27]. The definitions of PFD adhere to the relevant joint reports and cut‐offs for PFD adhere to previous studies except for faecal incontinence where all degrees of bother were included given the severity of this symptom [24, 25, 28, 29, 30, 31].

Three‐dimensional EVUS was carried out, with the women in dorsal lithotomy position, hips flexed and abducted, by one of two urogynaecologists (coauthor MHJ, and KF (see Acknowledgments)). The probe was introduced into the vagina in a neutral position. A BK Medical Flex Focus machine with two different probes, BK 8838 6–16 MHz and BK 2052 6‐16Mhz, was used (BK Medical, Burlington, MA, USA). While the BK 8838 probe has a built‐in automatic linear array 360° acquisition of 1440 two‐dimensional (2D) images of 0.25° each, BK 2052 has an internal automated motorised system allowing an acquisition range of 60 mm consisting of 300 transaxially aligned 2D images of 0.2 mm each. Both transducers allow 3D acquisition without any movement of the probe within the cavity. A set‐up of 9 MHz was used for both probes. Before the examinations commenced, two instructors experienced in conducting EVUS (coauthor ER, and MS (see Acknowledgments)) carried out investigator training and observation for 2 days.

The levator ani muscle was divided into three sections in accordance with published work by Rostaminia et al. [6]: puboperinealisis/puboanalis; puborectalis; and pubococcygeus/iliococcygeus, as shown in Figure S1A [1]. Each section was assessed bilaterally in its specific axial plane where the full length of muscle was visualised and scored on each side based on thickness and detachment from the pubic bone, as follows: 0 = no defect; 1 = minimal defect with ≤ 50% muscle loss; 2 = major defect with > 50% loss; and 3 = total absence. The scores of each subgroup on both sides were summed into a LAD score (0–18 points). This score was categorised as 0–6 (mild deficiency), 7–12 (moderate deficiency), and ≥ 13 (severe deficiency) points, as defined by Rostaminia et al. (Figure S1B) [6, 8]. Volumes were analysed if the pubic symphysis and levator ani muscle including all subgroups were visualised in an axial section, otherwise the volumes were excluded. Ultrasound volumes acquired with the 8838 probe were analysed as a primary choice; volumes acquired with the 2052 probe were only analysed if those acquired with the 8838 probe were deemed uninterpretable or were missing. The 8838 probe was selected as the primary choice because the contrast resolution is perceived as higher compared with the 2052 probe although neither probe has proven superior diagnostic abilities compared with the other [9, 32].

Volumes were analysed later in a blinded fashion by a primary assessor (MHJ). A maximum of 10 volumes were analysed per session. Where the LAD score was > 0 points the volume was assessed a second time on another day. In case of discrepancy, the volume was assessed on a third occasion All volumes with LAD score ≥ 6, or volumes that had discrepant values by the primary assessor, were assessed by a second assessor (ER), also in a blinded fashion. Thereafter the two assessors compared their assessments and agreed on a final score for each volume. The primary assessor (MHJ) had 9 years' experience in 3D EVUS assessment and has had repeated structured training in interpretation by two senior assessors (ER and MS). The secondary assessor (ER) had 12 years' experience in conducting 3D EVUS. Patients were not involved in the design of the study; however, the study has been inspired by patient prioritisation of research questions on maternal birth injuries [11, 33].

### 2.1 Sample Size

The present study is one of several reports from the POPRACT study, which had many explorative outcomes with unclear prevalence [17]. Although a formal sample size calculation was not performed a priori, we deemed that a sample size of approximately 200 participants would be sufficient to assess associations of clinical interest.

### 2.2 Statistical Analysis

Descriptive data are presented as numbers, proportions, means with standard deviations (SDs) and medians with interquartile ranges (IQRs). Associations between LAD score and various types of pelvic floor symptoms were evaluated using logistic regression models, estimating risk ratios (ORs) with 95% confidence intervals (CIs). Adjusted odds ratios (aORs) were obtained including potential confounding variables (age, body mass index [BMI], and mode of vaginal birth) in the model in addition to the risk factors of interest. Cut‐offs for LAD scores other than those previously described were used to investigate the optimal dichotomous categories of LAD score for various symptoms. Differences between groups were compared using a chi‐squared test for categorical variables, a *t*‐test for normally distributed continuous variables, and a Wilcoxon rank‐sum test for continuous variables that exhibited asymmetry. Data were analysed using Stata/SE version 16.0 (StataCorp LP, College Station, TX, USA), with a few exceptions: graph bars were created in SPSS version 29.0 (IBM Corp., Armonk, NY, USA) and forest plots were created in R version 4.3.1 (R Foundation for Statistical Computing, Vienna, Austria).

## 3 Results

Of the 706 women in the POPRACT cohort responding to the questionnaire at 1 year postpartum, 212 were identified as meeting the symptom criteria for cases and were invited to attend the examination (Figure S2). A total of 103 women underwent EVUS examination according to the protocol and fulfilled the inclusion criteria and were therefore included as cases. Among the 488 women who did not report significant symptoms, 87 were examined according to the protocol, met the inclusion criteria and were included as controls.

Participant characteristics including time to follow‐up and prevalence of pelvic floor symptoms are presented in Table 1. There were no differences between cases and controls regarding age, BMI or follow‐up time postpartum. The most common symptoms among cases were vaginal laxity and urinary incontinence, reported by 28% and 29% of cases, respectively. Two participants in the case group and three in the control group had an uninterpretable 3D volume acquired with the BK 8838 probe but had an interpretable volume acquired with BK 2052 probe. For these participants, the latter volume was analysed. No statistically significant differences in baseline characteristics were found between included women and women from the POPRACT cohort who were not included in the present study, except for delivery mode, which was expected given that Caesarean delivery was an exclusion criterion (Table S2).

**TABLE 1:** Characteristics of cases (*n* = 103) and controls (*n* = 87).

	Cases	Controls	*p*
	*n* (%)	*n* (%)	
**Age at delivery**			
Mean [SD] years	29.5 [3.9]^a^	28.4 [3.8]	0.08
Median [IQR] years	29 [27–32]	28 [26–31]	
≤ 25 years	16 (16)	18 (21)	0.45
26–30 years	49 (48)	46 (53)	
31–35 years	31 (30)	18 (21)	
> 35 years	7 (7)	5 (6)	
Missing	0	0	
**BMI at 1 year postpartum**			
Mean [SD] kg/m^2^	25.2 [4.8]	24.4 [4.7]	
Median [IQR] kg/m^2^	24.3 [21.9–27.0]	23.5 [21.5–26.9]	0.23
≤ 25 kg/m^2^	57 (57)	57 (66)	0.49
25.1–30 kg/m^2^	28 (28)	22 (25)	
30.1–35 kg/m^2^	11 (11)	6 (7)	
> 35.1 kg/m^2^	5 (5)	2 (2)	
Missing	2	0	
**Education**			
9–< 12 years	1 (1)	1 (1)	0.99
12 years	27 (28)	24 (28)	
University	70 (71)	61 (71)	
Missing	5	1	
**Smoking**	5 (5)	1 (1)	0.13
No	93 (95)	85 (99)	
Missing	5	1	
**Delivery mode**			
Spontaneous vaginal delivery	87 (84)	72 (83)	0.75
Vacuum extraction	16 (16)	15 (17)	
Missing	0	0	
**Follow‐up time postpartum**			
Mean [SD] days	442 [42]	447 [49]	
Median [IQR]	434 [414–469]	432 [416–494]	0.74
Symptoms of pelvic floor dysfunction			
**Urinary symptoms**			n/a
**Urinary incontinence** ^**a**^	29 (28)	0	
No	74 (72)	87 (100)	
Missing	0	0	
**Urinary leakage during sex** ^**c**^	4 (4)	0	
No	87 (96)	78 (100)	
Missing	12	9	
**Fear of urinary leakage during sex** ^**c**^	19 (21)	0	
No	72 (79)	77 (100)	
Missing	12	10	
**Prolapse and other vaginal symptoms**			n/a
**Vaginal bulging** ^**d**^	34 (33)	0	
No	68 (67)	87 (100)	
Missing	1	0	
**Digitation/splinting** ^**a**^	16 (16)	0	
No	86 (84)	86 (100)	
Missing	1	1	
**Vaginal chafing** ^**e**^	3 (3)	0	
No	100 (97)	87 (100)	
Missing	0	0	
**Lifting vaginal wall for urination** ^**d**^	2 (2)	0	
No	101 (98)	87 (100)	
Missing	0	0	
**Sex avoidance due to vaginal bulging** ^**c**^	13 (14)	0	
No	79 (86)	77 (100)	
Missing	11	10	
**Vaginal laxity** ^**f**^	30 (29)	0	
No	73 (71)	87 (100)	
Missing	0	0	
**Anal incontinence symptoms**			n/a
**Faecal incontinence** ^**f**^	18 (18)	0	
No	84 (82)	87 (100)	
Missing	1	0	
**Flatus incontinence** ^**b**^	19 (18)	0	
No	84 (82)	87 (100)	
Missing	0	0	

Abbreviations: BMI, body mass index; IQR, interquartile range; n/a, not applicable to compare symptoms in cases and controls; SD, standard deviation.

^a^ Numbers in square brackets show SD or IQR.

^b^ Reported to bother the participant ‘moderately’ or ‘quite a bit’.

^c^ Reported to occur ‘always’, ‘often’ or ‘sometimes’.

^d^ Reported to occur ‘often’ or ‘sometimes’.

^e^ Reported to occur ‘often’.

^f^ Any degree of bother or frequency.

**FIGURE 1:**
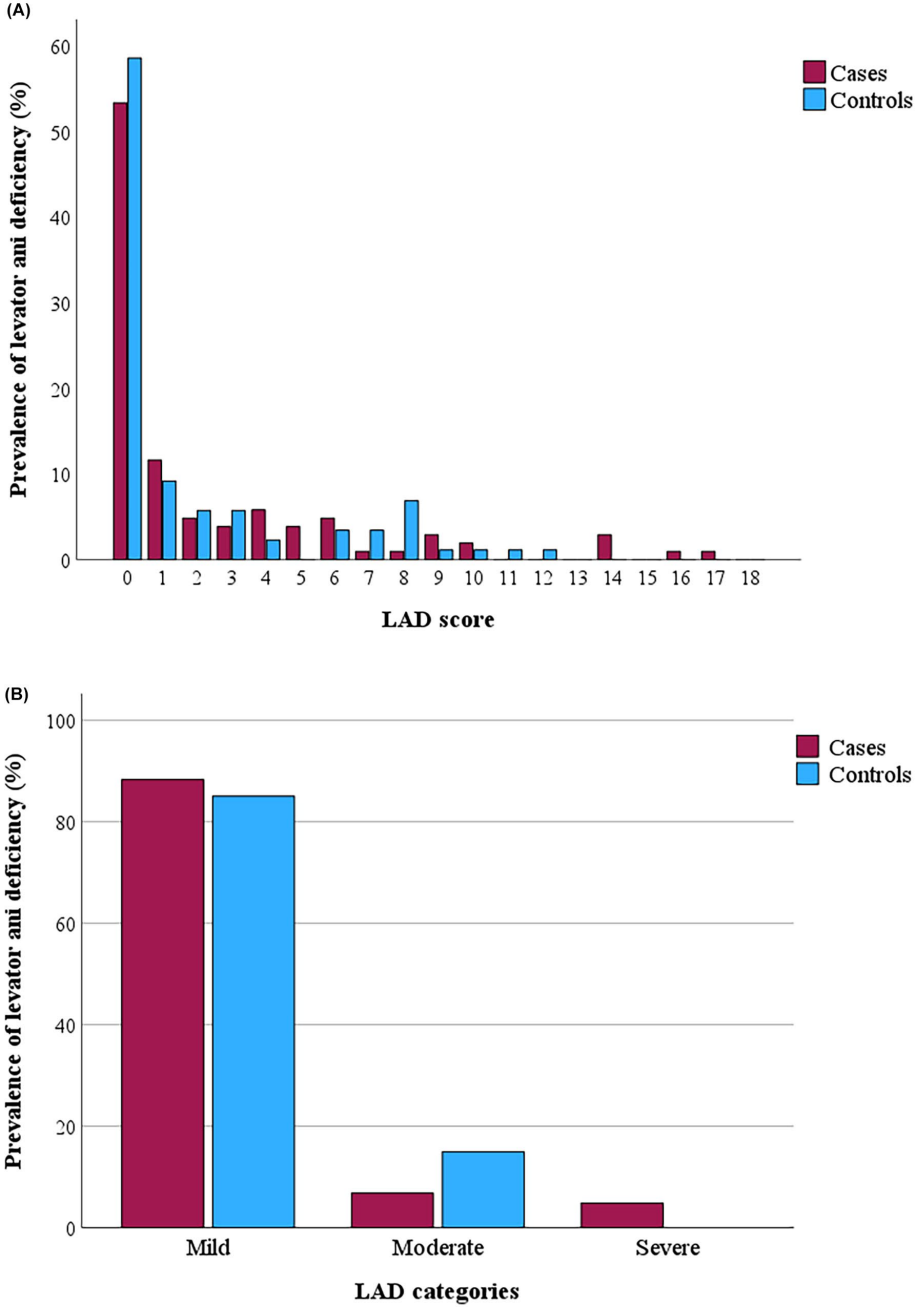
Prevalence of levator ani deficiency (LAD) among cases and controls, presented as LAD score (A) and LAD categories (B).

Overall, the levator ani deficiency rate was low. 53% in the case group, and 58% in the control group had a LAD score of 0 (Figure 1A). Among cases, seven participants had moderate (7%) and five had severe levator ani deficiency (5%), whereas among controls, 13 had moderate levator ani deficiency (15%) and none had severe levator ani deficiency (Figure 1B). The distribution of levator ani deficiency categories differed significantly between cases and controls (*p* = 0.027).

An increased LAD score was significantly associated with urinary incontinence, especially at low scores (Table 2 and Figure S3). The risk for incontinence was increased when cut‐offs were set at between ≥ 1 and ≥ 4 points (Figure S3). Additionally, an increased LAD score was significantly associated with vaginal laxity (Table 2). The risk of vaginal laxity was increased when the cut‐off for LAD was set at between ≥ 8 points and ≥ 14 points (Figure S4). The two participants with a LAD score ≥ 15 points were cases but neither reported urinary incontinence or vaginal laxity, which explains why no cut‐off for LAD ≥ 15 was estimated.

**TABLE 2:** Association between levator ani deficiency score and pelvic floor dysfunction. Age at delivery, body mass index and mode of vaginal birth were included as potential confounders and were mutually adjusted for.

	OR (95% CI)	*n*	aOR (95% CI)	*n*
Urinary symptoms				
Urinary incontinence^a^	1.10 (1.00, 1.21)*	190	1.11 (1.00, 1.22)*	188
Urinary leakage during sex^b^	0.90 (0.61, 1.33)*	169	0.85 (0.52, 1.39)^c^	168
Fear of UI during sex^b^	0.90 (0.75, 1.08)	168	0.91 (0.75, 1.10)	167
Prolapse and other vaginal symptoms				
Vaginal bulging^d^	0.93 (0.82, 1.06)	189	0.92 (0.81, 1.05)	187
Digitation/splinting^a^	1.06 (0.94, 1.20)	188	1.09 (0.95, 1.24)	186
Vaginal chafing^e^	0.91 (0.59, 1.41)	190	0.91 (0.55, 1.48)^f^	188
Lifting vaginal wall for urination^c^	NE^g^		NE^g^	
Sex avoidance due to vaginal bulging^b^	1.02 (0.88, 1.19)	169	1.01 (0.86, 1.19)	168
Vaginal laxity^g^	1.14 (1.04, 1.25)*	190	1.14 (1.03, 1.25)*	188
Anal incontinence symptoms				
Faecal incontinence^h^	1.03 (0.91, 1.17)	189	1.02 (0.89, 1.17)	187
Flatus incontinence^a^	1.05 (0.94, 1.18)	190	1.05 (0.94, 1.19)	188

Abbreviations: aOR, adjusted odds ratio; CI, confidence interval; NE, not estimated; OR, odds ratio; UI, urinary incontinence.

* *p* < 0.05.

^a^ Reported to bother the participant ‘moderately’ or a ‘quite a bit’.

^b^ Reported to occur ‘always’, ‘often’ or ‘sometimes’.

^c^ Not adjusted for vaginal delivery mode because there was no participant with vacuum extraction assisted delivery and the outcome studied.

^d^ Reported to occur ‘often’ or ‘sometimes’.

^e^ Reported to occur ‘often’.

^f^ Not adjusted for mode of vaginal birth because of nonconvergence when vaginal delivery mode was included in the model.

^g^ There were no participants with this symptom and LAD score > 0.

^h^ Any degree of bother or frequency.

When LAD score was categorised according to previous literature [6], severe levator ani deficiency was associated with vaginal laxity (Table 3).

**TABLE 3:** Association between levator ani deficiency (LAD) category and symptoms of pelvic floor dysfunction (*N* = 190). Age at delivery, body mass index (BMI) and mode of vaginal birth were included as potential confounders and were mutually adjusted for.

	Yes *n* (%)	No *n* (%)	Missing	OR (95% CI)	*n*	aOR	*n*
Urinary symptoms							
Urinary incontinence^a^					190		188
Mild LAD	24 (15)	141 (85)	0	1.00		1.00	
Moderate LAD	3 (15)	17 (85)	0	1.04 (0.28, 3.81)		1.06 (0.28, 3.96)	
Severe LAD	2 (40)	3 (60)	0	3.92 (0.62, 24.68)		3.98 (0.61, 25.86)	
Urinary leakage during sex^b^							
Mild LAD	4 (3)	142 (97)	19		—		—
Moderate LAD	0	19 (100)	1	NE		NE	
Severe LAD	0	4 (100)	1	NE		NE	
Fear of UI during sex^b^					164		163
Mild LAD	18 (12)	127 (88)	20	1.00		1.00	
Moderate LAD	1 (5)	18 (95)	1	0.39 (0.05, 3.12)		0.43 (0.05, 3.45)	
Severe LAD	0	4 (100)	1	NE^c^		NE^c^	
Prolapse or other vaginal symptoms							
Vaginal bulging^d^					189		187
Mild LAD	31 (19)	134 (81)	0	1.00		1.00	
Moderate LAD	2 (10)	18 (90)	0	0.48 (0.11, 2.18)		0.46 (0.10, 2.08)	
Severe LAD	1 (25)	3 (75)	1	1.44 (0.14, 14.32)		1.22 (0.12, 12.27)	
Digitation/splinting^a^					188		186
Mild LAD	14 (9)	150 (91)	1	1.00		1.00	
Moderate LAD	1 (5)	18 (95)	1	0.60 (0.07, 4.80)		0.68 (0.08, 5.64)	
Severe LAD	1 (20)	4 (80)	0	2.68 (0.28, 25.64)		4.58 (0.44, 47.94)	
Vaginal chafing^e^							
Mild LAD	3 (2)	162 (98)	0				
Moderate LAD	0 (0)	20 (100)	0	NE^f^		NE^f^	
Severe LAD	0 (0)	5 (100)	0	NE^f^		NE^f^	
Lifting vaginal wall for urination^d^							
Mild LAD	2 (1)	163 (99)	0				
Moderate LAD	0 (0)	20 (100)	0	NE^b^		NE^b^	
Severe LAD	0 (0)	5 (100)	0	NE^b^		NE^b^	
Sex avoidance due to vaginal bulging^b^					169		168
Mild LAD	11 (8)	135 (92)	19	1.00		1.00	
Moderate LAD	1 (5)	18 (95)	1	0.68 (0.08, 5.60)		0.69 (0.08, 5.75)	
Severe LAD	1 (25)	3 (75)	1	4.09 (0.39, 42.69)		5.38 (0.47, 61.88)	
Vaginal laxity					188		188
Mild LAD	23 (14)	142 (86)	0	1.00		1.00	
Moderate LAD	3 (15)	17 (85)	0	1.09 (0.3, 4.01)		1.02 (0.27, 3.86)	
Severe LAD	4 (80)	1 (20)	0	24.7 (2.64, 230.85)*		28.64 (2.97, 276.16)*	
Anal incontinence symptoms							
Faecal incontinence					189		187
Mild LAD	15 (9)	149 (91)	1	1.00		1.00	
Moderate LAD	2 (10)	18 (90)	0	1.10 (0.23, 5.22)		1.17 (0.24, 5.74)	
Severe LAD	1 (20)	4 (80)	0	2.48 (0.26, 23.67)		3.11 (0.31, 31.49)	
Flatus incontinence^a^					190		188
Mild LAD	16 (10)	149 (90)	0	1.00			
Moderate LAD	1 (5)	19 (95)	0	0.49 (0.06, 3.91)		0.49 (0.06, 3.96)	
Severe LAD	2 (40)	3 (60)	0	6.21 (0.96, 39.96)		6.29 (0.94, 42.25)	

Abbreviations: aOR, adjusted odds ratio; CI, confidence interval; NE, not estimated; OR, odds ratio; UI, urinary incontinence.

* *p <* 0.05.

^a^ Reported to bother the participant ‘moderately’ or a ‘quite a bit’.

^b^ Reported to occur ‘always’, ‘often’ or ‘sometimes’.

^c^ There were no participants in this category.

^d^ Reported to occur ‘often’ or ‘sometimes’.

^e^ Reported to occur ‘often’.

^f^ There were no participants in this category.

## 4 Discussion

### 4.1 Main Findings

This nested case–control study of primiparous women with or without significant pelvic floor symptoms 1 year postpartum found overall low LAD scores, with the majority of the women having no levator ani deficiency at all. Increased LAD scores were associated with urinary incontinence and vaginal laxity. Severe levator ani deficiency was associated with vaginal laxity.

### 4.2 Strengths and Limitations

There are several strengths to our study. To the best of our knowledge, this is the first study to evaluate the association between levator ani deficiency assessed using EVUS, and a wide range of pelvic floor symptoms in a large sample of primiparous women examined 1 year postpartum. Data on pelvic floor symptoms were prospectively collected with questions from validated questionnaires. The EVUS volumes were analysed in a blinded fashion using the LAD scoring system, which is a validated and detailed scoring system for the assessment of levator defects [8, 9]. Furthermore, the included cases and controls appear to be representative of the symptomatic and asymptomatic study participants of the cohort from which they were drawn. Also, we regard the information from participants about their levator ani status prior to answering the questionnaire as limited; their response should therefore not have been biased by previous information they had provided.

In terms of limitations, we acknowledge that the observational design and the exploratory aim of the study imply that no extensive inferences about causality should be drawn from our results. The difficulties to recruit women to the control group made it less feasible to match controls, which is a limitation. Even though our sample appears to be representative of the mother cohort, the generalisability may be limited to countries with a similar population and setting for childbirth and postpartum follow‐up. Despite a relatively large sample size, there were few participants with a high LAD score. No formal sample size calculation was performed when planning the study, given the unknown incidence of LAD and the multiple outcomes of the whole POPRACT study. Therefore, the absence of association between LAD score and some pelvic floor symptoms may be due to limited power of the study. Furthermore, EVUS assesses morphology of the levator ani muscle but does not assess the dynamic function of the levator. The morphology of levator ani muscle, assessed using EVUS, has been described as moderately predictive of muscle strength as assessed by the Modified Oxford Scale [10].

No core outcome sets for PFD were available when this study was planned. We adhered to the definitions of PFD in relevant joint reports but these did not include any cut‐offs based on frequency or bother, as we have used [28, 29]. We consider the use of cut‐offs in the present study important in terms of not diluting our case group with participants with insignificant symptoms. The lack of a core outcome set for PFD makes comparison of study results difficult, and there is an urgent need to define a core outcome set in this field.

### 4.3 Interpretation

We are not aware of any previous study examining the relationship between levator ani deficiency assessed using EVUS, and urinary incontinence, but the association between levator defects, visualised by either MRI or TPUS, and urinary incontinence has been studied repeatedly. A systematic review and meta‐analysis of six such studies, five of which used TPUS, concluded that there is no relationship [34]. Still, some studies report an association [15, 35]. In the present study, urinary incontinence was associated with levator ani deficiency based on cut‐offs at lower LAD scores. The ability of EVUS to visualise even such minor levator defects including the puboperinealis/puboanalis portions might explain why we found an association while most studies using TPUS did not [34]. The puboperinealis/puboanalis portion supports the midurethra through the perineal membrane, and weakening of this support is a possible pathophysiological explanation for our finding [36].

We found that severe levator ani deficiency was associated with vaginal laxity, which is consistent with previous studies assessing levator avulsion using TPUS [13, 14]. Our findings do not tell whether this sensation was bothersome, since the answer option was only whether or not the symptom occurred. We found no association between levator ani deficiency and avoidance of sex due to vaginal bulging, perhaps because of limited sample size. The association between levator ani deficiency and sexual function was not explored in the present study, which is a limitation. Previous studies found no association between wide vagina and sexual function, nor between levator avulsion and sexual function, but concluded that this is a poorly investigated field [13, 14]. Therefore, we suggest that future research on levator ani deficiency should include aspects of sexual function.

A previous study by Rostaminia et al. found an association between degree of levator ani deficiency assessed by EVUS, and anatomical prolapse stage while we found no association between levator ani deficiency and vaginal bulging [6]. Their study sample was included in a urogynaecology clinic setting and had a higher mean age and plausibly a higher prevalence of uterovaginal prolapse compared with our cohort, which may at least partly explain why they found an association and we did not [6].

How to counsel and treat women with levator defects is a new avenue of research. The present study provides insight into which symptoms are associated with levator defects. Such information is requested by both affected women and healthcare providers, according to working group discussions on prioritised research [11]. Increased knowledge on the symptoms of levator injuries may guide patients and healthcare providers alike regarding when to seek health care for a diagnosis. The current literature is unclear about whether conservative treatment including physiotherapy is helpful in this area but, given the reduced muscle strength observed in previous studies, levator defects may impair the effect of pelvic floor exercises on, for example, stress urinary incontinence [37, 38]. Since levator ani deficiency was associated with urinary incontinence in the present study, we suggest that future studies should address whether pelvic floor exercises are effective against urinary incontinence in women with levator defects. The fact that a considerable number of women with moderate levator deficiency were asymptomatic could be emphasised when counselling women who fear levator injury.

## 5 Conclusions

This nested case–control study indicates that levator ani deficiency may be associated with both urinary incontinence and vaginal laxity and that the risk of each symptom may increase at certain LAD score cut‐offs, and merits further studies. While the risk of urinary incontinence increased already with minor levator ani deficiency, the risk of vaginal laxity only increased when the levator ani deficiency was extensive. Defects of the most medial levator ani muscle portions offer a possible pathophysiological explanation for the observed increase in urinary incontinence. A better understanding of the symptomatology of levator ani deficiency may guide patients and healthcare providers alike regarding when to seek health care and how to provide the affected women with a constructive treatment plan.

## Author Contributions

M.H.J.: project development, data collection and management, data analysis, manuscript writing. S.B.W.: data analysis, manuscript editing. E.R.: project development, data collection, data analysis, manuscript editing.

## Ethics Statement

The study was approved by the Regional Ethical Review Board in Stockholm on 21 February 2014 (registration number: 2014/124–32). All participants provided written informed consent upon recruitment and were informed that they could withdraw from the study at any time, without having to give a reason. The study was performed in line with the principles of the Declaration of Helsinki.

## Conflicts of Interest

The authors declare no conflicts of interest.

## Supporting information


Data S1.

## Data Availability

The data that support the findings of this study are available from the corresponding author upon reasonable request.
