# Supplementary material for: Levator Ani Deficiency and Pelvic Floor Dysfunction 1 Year Postpartum: A Prospective Nested Case–Control Study
Source: BJOG. 2024 Dec 3;132(5):596–605. doi: 10.1111/1471-0528.18036 (PMC11879914; doi:10.1111/1471-0528.18036)

**Table S1.** Questions on pelvic floor dysfunction.

| **Questions** | **Answer options** |
| --- | --- |
| 1. Do you occasionally experience urinary leakage?^a^ | - No - Yes |
| If “Yes”, how much does it bother you?^b^ | - Not at all - Somewhat - Moderately - Quite a bit |
| 1. Do you ever have to push on the vagina or around the rectum to have or complete a bowel movement?^b^ | - No - Yes |
| If “Yes”, how much does it bother you?^b^ | - Not at all - Somewhat - Moderately - Quite a bit |
| 1. Do you usually lose gas from the rectum beyond your control? | - No - Yes |
| If “Yes”, how much does it bother you?^b^ | - Not at all - Somewhat - Moderately - Quite a bit |
| 1. Do you usually lose stool beyond your control if your stool is well formed?^b^ | - No - Yes |
| If “Yes”, how much does it bother you?^b^ | - Not at all - Somewhat - Moderately - Quite a bit |
| 1. Do you usually lose stool beyond your control if your stool is loose?^b^ | - No - Yes |
| If “Yes”, how much does it bother you?^b^ | - Not at all - Somewhat - Moderately - Quite a bit |
| 1. Do you have a sensation of tissue protruding from your vagina (vaginal bulge)?^c^ | - Yes, often - Sometimes - Infrequently - No, never |
| 1. Do you suffer from chafing in your vagina/vulva?^c^ | - Yes, often - Sometimes - Infrequently - No, never |
| 1. Do you have to lift the anterior vaginal wall to start or complete voiding?^c^ | - Yes, often - Sometimes - Infrequently - No, never |
| 1. Are you sexually active? | - Yes - No   If “No”, please proceed to question #13 |
| 1. Are you incontinent of urine (do you leak urine) with sexual activity?^b^ | - Always - Usually - Sometimes - Seldom - Never |
| 1. Does fear of incontinence (either stool or urine) restrict your sexual activity?^b^ | - Always - Usually - Sometimes - Seldom - Never |
| 1. Do you avoid sexual intercourse because of bulging of the vagina (prolapse)?^b^ | - Always - Usually - Sometimes - Seldom - Never |
| 1. Do you have any other pelvic floor symptoms that are not mentioned above? (Several options may be chosen) | - Impaired ability to contract the pelvic floor - Gas in vagina during sexual intercourse or physical training - Sensation of impaired genital feeling - Sensation of wide vagina - Feeling of insufficient support of the rear vaginal wall towards the rectum - Other … (please specify) |

^a^Luthander C, Emilsson T, Ljunggren G, Hammarstrom M. A questionnaire on pelvic floor dysfunction postpartum. Int Urogynecol J. 2011;22(1):105–13.

^b^Teleman P, Stenzelius K, Iorizzo L, Jakobsson U. Validation of the Swedish short forms of the Pelvic Floor Impact Questionnaire (PFIQ-7), Pelvic Floor Distress Inventory (PFDI-20) and Pelvic Organ Prolapse/Urinary Incontinence Sexual Questionnaire (PISQ-12). Acta Obstet Gynecol Scand. 2011;90(5):483–7.

^c^Tegerstedt G, Miedel A, Maehle-Schmidt M, Nyren O, Hammarstrom M. A short-form questionnaire identified genital organ prolapse. J Clin Epidemiol. 2005;58(1):41–6.

**Table S2.** Characteristics of included women with significant symptoms (cases, n=103), women with significant symptoms not included in the present study (n=109), included women without significant symptoms (controls, n=87), and women without significant symptoms not included in the present study (n=401).

|  | Significant symptoms | |  | No significant symptoms | | |  | |
| --- | --- | --- | --- | --- | --- | --- | --- | --- |
|  | Cases | Not  included |  | Controls | Not included |  | |  |
|  | n (%) | n (%) | *p* | n (%) | n (%) | *p* | |  |
| **Age at delivery** |  |  |  |  |  |  | |  |
| Mean [SD] years | 29.4 [3.9]^a^ | 29.2 [3.7] | 0.63 | 28.4 [3.8] | 28.8 [4.0] | 0.41 | |  |
| Median [IQR] years | 29 [27– 32] | 28 [27–32] |  | 28 [26– 31] | 29 [26–31] |  | |  |
| ≤25 years | 16 (16) | 18 (17) | 0.81 | 18 (21) | 74 (18) | 0.86 | |  |
| 26–30 years | 49 (48) | 57 (53) |  | 46 (53) | 203 (51) |  | |  |
| 31–35 years | 31 (30) | 29 (26) |  | 18 (21) | 96 (24) |  | |  |
| >35 years | 7 (7) | 5 (5) |  | 5 (6) | 28 (7) |  | |  |
| Missing | 0 | 0 |  | 0 | 0 |  | |  |
| **BMI at 1 year postpartum** |  |  |  |  |  |  | |  |
| Mean [SD] kg/m^2^ | 25.2 [4.8] | 25.3 [5.4] |  | 24.4 [4.7] | 25.1 [4.9] |  | |  |
| Median [IQR] kg/m^2^ | 24.3 [21.9– 27.0] | 23.6 [21.1–27.7] | 0.68 | 23.5 [21.5–26.9] | 24.2 [21.7–27.5] | 0.19 | |  |
| ≤25 kg/m^2^ | 57 (57) | 67 (62) | 0.64 | 57 (66) | 227 (58) | 0.50 | |  |
| 25.1–30 kg/m^2^ | 28 (28) | 22 (20) |  | 22 (25) | 109 (28) |  | |  |
| 30.1–35 kg/m^2^ | 11 (11) | 12 (11) |  | 6 (7) | 41 (10) |  | |  |
| >35.1 kg/m^2^ | 5 (5) | 7 (6) |  | 2 (2) | 16 (4) |  | |  |
| Missing | 2 | 1 |  | 0 | 8 |  | |  |
| **Education** |  |  |  |  |  | 0.50 | |  |
| 9–<12 years | 1 (1) | 0 | 0.14 | 1 (1) | 7 (2) |  | |  |
| 12 years | 27 (28) | 40 (39) |  | 24 (28) | 131 (34) |  | |  |
| University | 70 (71) | 62 (61) |  | 61 (71) | 249 (64) |  | |  |
| Missing | 5 | 7 |  | 1 | 14 |  | |  |
| **Smoking** | 5 (5) | 1 (1) | 0.09 | 1 (1) | 7 (2) | 0.67 | |  |
| No | 93 (95) | 102 (99) |  | 85 (99) | 380 (98) |  | |  |
| Missing | 5 | 6 |  | 1 | 14 |  | |  |
| **Delivery mode** |  |  |  |  |  | <0.01 | |  |
| Spontaneous vaginal delivery | 87 (84) | 67 (62) | <0.01 | 72 (83) | 258 (64) |  | |  |
| Vacuum extraction | 16 (16) | 19 (18) |  | 15 (17) | 64 (16) |  | |  |
| Elective Caesarean section | 0 | 7 (6) |  | 0 | 31 (8) |  | |  |
| Acute Caesarean section | 0 | 15 (14) |  | 0 | 48 (12) |  | |  |
| Missing | 0 | 1 |  | 0 | 0 |  | |  |
| **Symptoms of pelvic floor dysfunction** |  |  |  |  |  |  | |  |
| **Urinary symptoms** |  |  |  |  |  |  | |  |
| **Urinary incontinence^b^** | 29 (28) | 28 (26) | 0.72 | 0 | 0 | NE | |  |
| No | 74 (72) | 80 (74) |  | 87 (100) | 400 (100) |  | |  |
| Missing | 0 | 1 |  | 0 | 1 |  | |  |
| **Urinary leakage during sex^c^** | 4 (4) | 10 (11) | 0.09 | 0 | 0 | NE | |  |
| No | 87 (96) | 80 (89) |  | 78 (100) | 367 (100) |  | |  |
| Missing | 12 | 19 |  | 9 | 34 |  | |  |
| **Fear of urinary leakage**  **during sex^c^** | 19 (21) | 24 (27) | 0.36 | 0 | 0 | NE | |  |
| No | 72 (79) | 66 (73) |  | 77 (100) | 367 (100) |  | |  |
| Missing | 12 | 19 |  | 10 | 34 |  | |  |
| **Prolapse and other vaginal**  **symptoms** |  |  |  |  |  |  | |  |
| **Vaginal bulging^d^** | 34 (33) | 86 (80) | 0.04* | 0 | 0 | NE | |  |
| No | 68 (67) | 22 (20) |  | 87 (100) | 399 (100) |  | |  |
| Missing | 1 | 1 |  | 0 | 2 |  | |  |
| **Digitation/splinting^b^** | 16 (16) | 9 (8) | 0.10 | 0 | 0 | NE | |  |
| No | 86 (84) | 100 (92) |  | 86 (100) | 401 (100) |  | |  |
| Missing | 1 | 0 |  | 1 | 0 |  | |  |
| **Vaginal chafing^e^** | 3 (3) | 9 (8) | 0.09 | 0 | 0 | NE | |  |
| No | 100 (97) | 100 (92) |  | 87 (100) | 399 (100) |  | |  |
| Missing | 0 | 0 |  | 0 | 2 |  | |  |
| **Lifting vaginal wall for urination^d^** | 2 (2) | 2 (2) | 0.95 | 0 | 0 | NE | |  |
| No | 101 (98) | 107 (98) |  | 87 (100) | 399 (100) |  | |  |
| Missing | 0 | 0 |  | 0 | 2 |  | |  |
| **Sex avoidance due to vaginal**  **bulging^c^** | 13 (14) | 9 (10) | 0.39 | 0 | 0 | NE | |  |
| No | 79 (86) | 81 (90) |  | 77 (100) | 369 (100) |  | |  |
| Missing | 11 | 19 |  | 10 | 32 |  | |  |
| **Vaginal laxity^f^** | 30 (29) | 29 (27) | 0.68 | 0 | 0 | NE | |  |
| No | 73 (71) | 80 (74) |  | 87 (100) | 399 (100) |  | |  |
| Missing | 0 | 0 |  | 0 | 2 |  | |  |
| **Anal incontinence symptoms** |  |  |  |  |  |  | |  |
| **Faecal incontinence^f^** | 18 (18) | 22 (20) | 0.64 | 0 | 0 | NE | |  |
| No | 84 (82) | 87 (80) |  | 87 (100) | 397 (100) |  | |  |
| Missing | 1 | 0 |  | 0 | 4 |  | |  |
| **Flatus incontinence^b^** | 19 (18) | 22 (20) | 0.75 | 0 | 0 | NE | |  |
| No | 84 (82) | 87 (80) |  | 87 (100) | 400 (100) |  | |  |
| Missing | 0 | 0 |  | 0 | 1 |  | |  |

**p*<0.05; BMI = body mass index; IQR = interquartile range; NE = not estimated; SD = standard deviation.

^a^Numbers in square brackets refer to standard deviation (SD) or interquartile range (IQR); ^b^reported to bother the participant “moderately” or a “quite a bit”; ^c^reported to occur always, often or sometimes; ^d^reported to occur often or sometimes; ^e^reported to occur often; ^f^any degree of bother or frequency.**Figure S1.** Levator ani subdivisions (A) and scoring system (B). The puboperinealis/puboanalis (PP/PA), puborectalis (PR), and pubococcygeus/iliococcygeus (PV) are shown in (A). (B) The scoring system in one of the three subdivisions of the levator ani muscle (puboperineal/puboanalis muscle). Source: Rotstein E et al. Three-dimensional endovaginal ultrasound assessment using the levator ani deficiency score in primiparas: a replication study. Acta Obstet Gynecol Scand. 2023 Sep;102(9):1236–42. Reproduced with permission under the Creative Commons Licence.


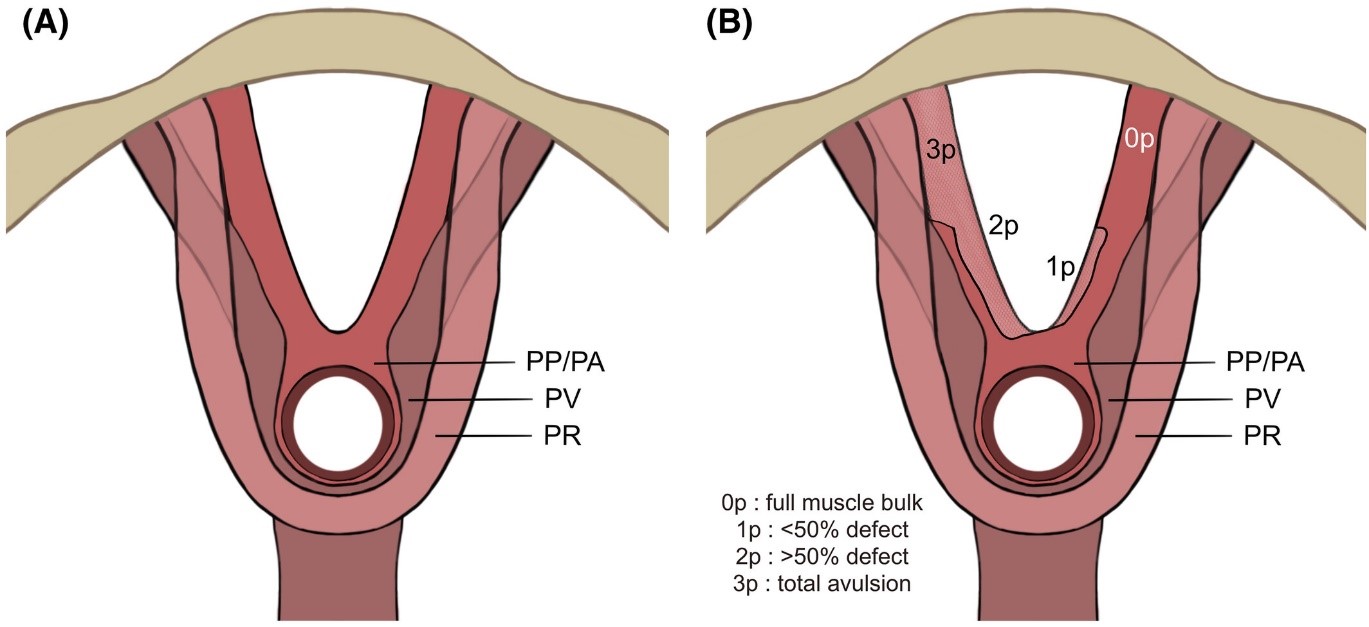


**Figure S2.** Flowchart of the study sample.


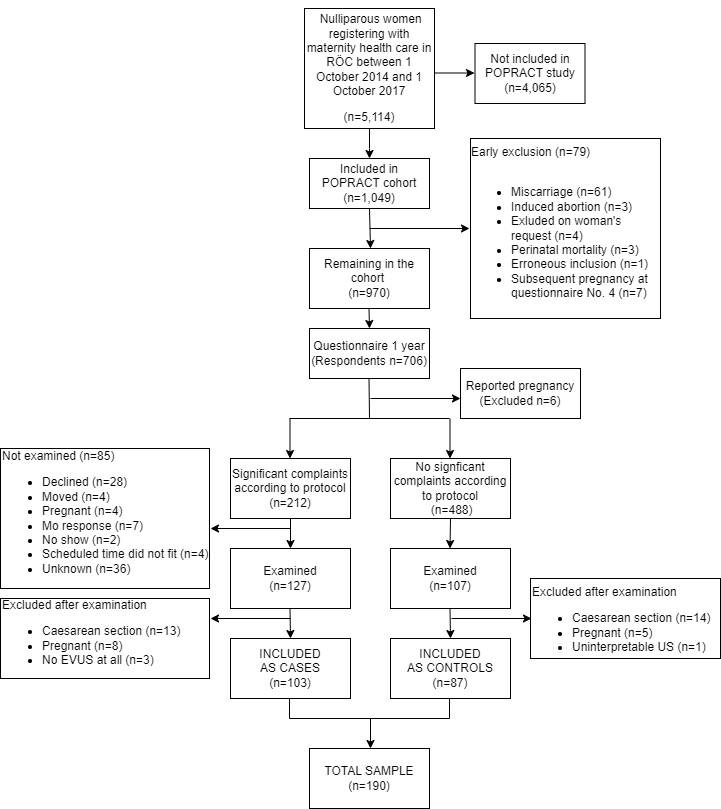


EVUS = three-dimensional endovaginal ultrasound; POPRACT = Pelvic Floor in Pregnancy and Childbirth; RÖC = Region Örebro County; US = ultrasound.

**Figure S3.** Adjusted odds ratios with 95% confidence intervals for the association between urinary incontinence and levator ani deficiency, using different cut-offs for levator ani deficiency score (LAD score).


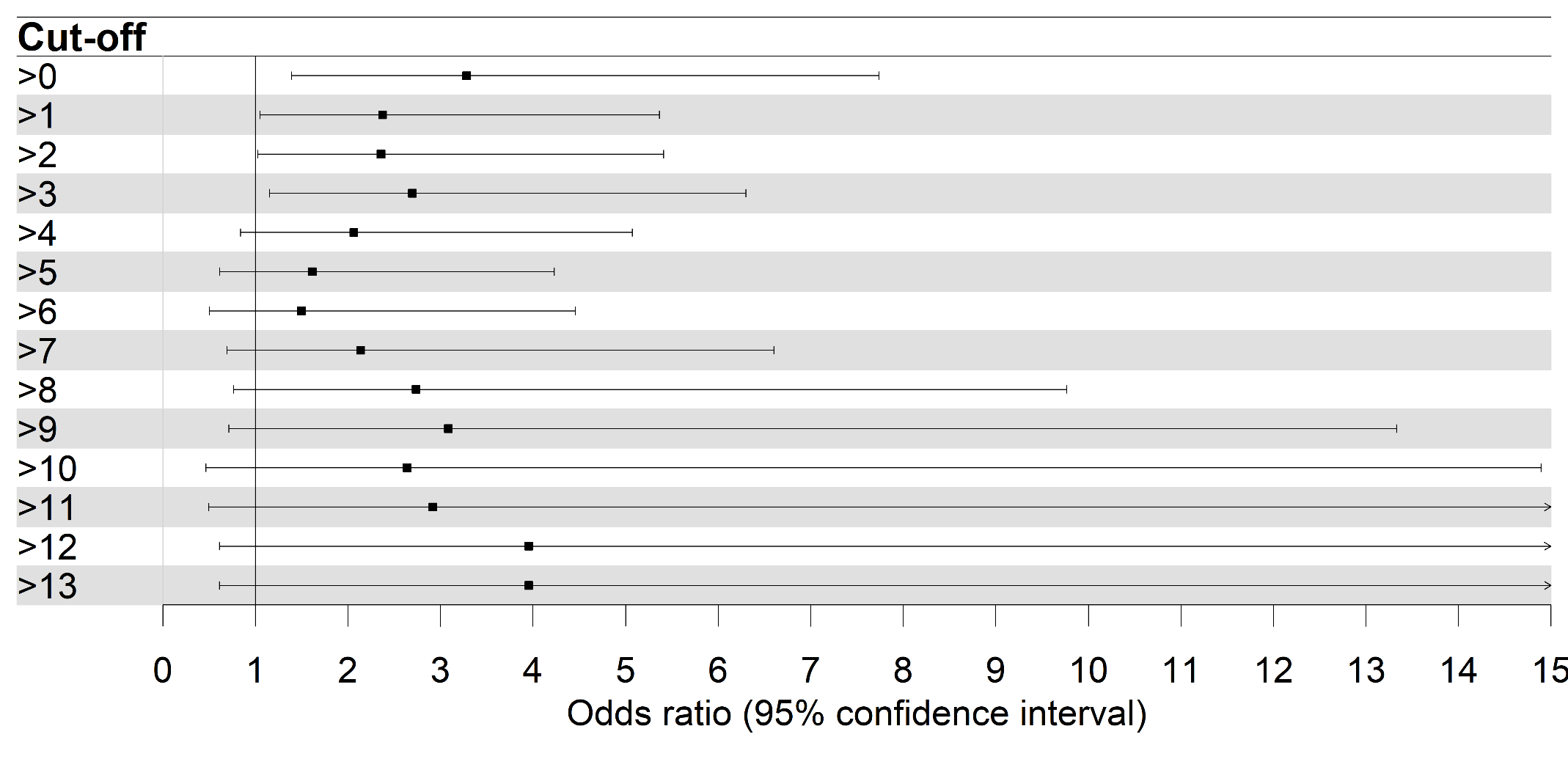


**Figure S4.** Adjusted odds ratios with 95% confidence intervals for the association between vaginal laxity and levator ani deficiency, using different cut-offs for levator ani deficiency score (LAD score).


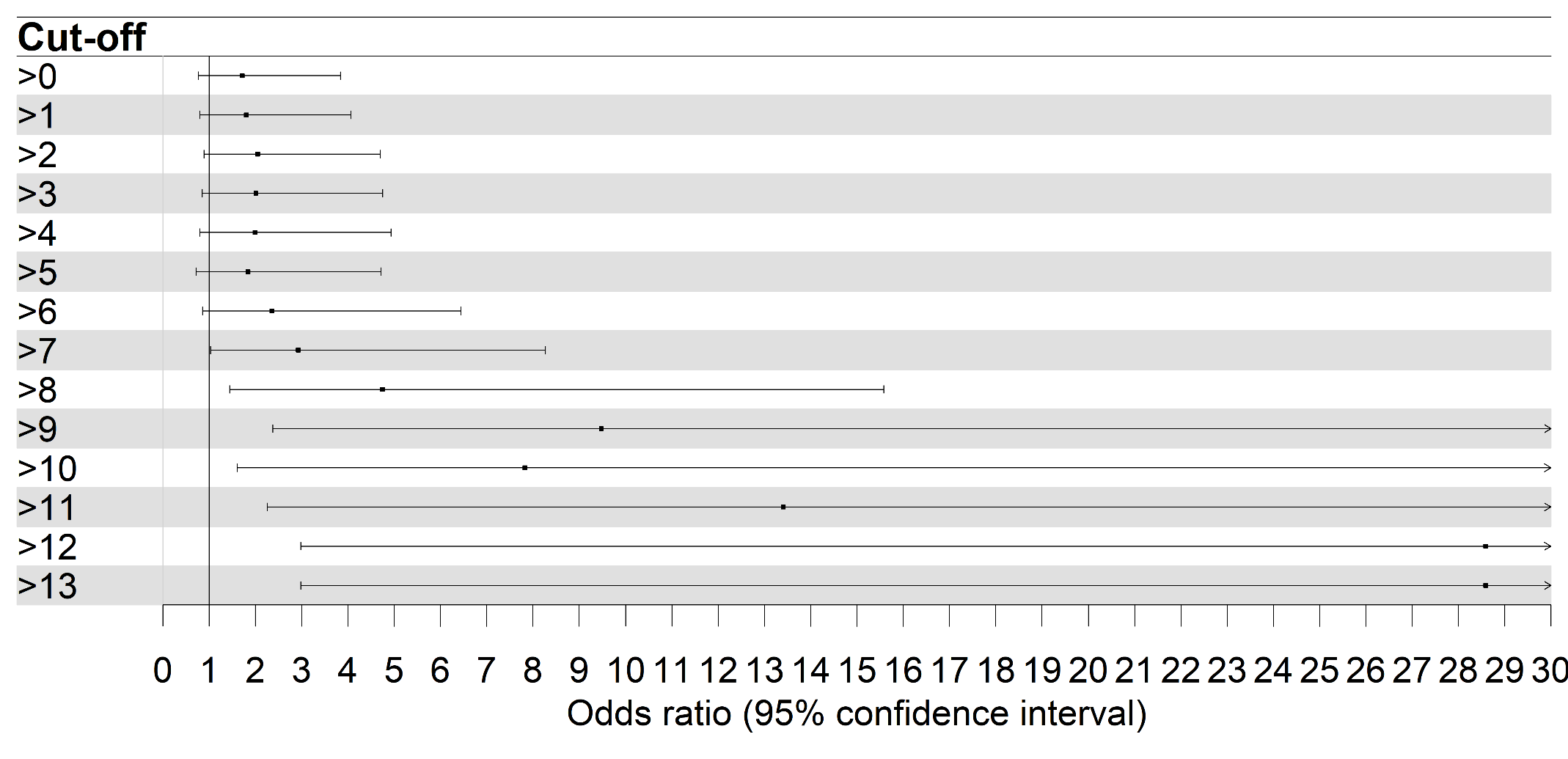

Supplement: Supplementary file 1 — Data S1. [file BJO-132-596-s001.docx]
